# Computational Studies towards the Identification of Novel Rhodopsin-Binding Compounds as Chemical Chaperones for Misfolded Opsins

**DOI:** 10.3390/molecules25214904

**Published:** 2020-10-23

**Authors:** Gaia Pasqualetto, Martin Schepelmann, Carmine Varricchio, Elisa Pileggi, Caroline Khogali, Siân R. Morgan, Ian Boostrom, Malgorzata Rozanowska, Andrea Brancale, Salvatore Ferla, Marcella Bassetto

**Affiliations:** 1 School of Pharmacy and Pharmaceutical Sciences, Cardiff University, Cardiff CF10 3NB, UK; PasqualettoG@cardiff.ac.uk (G.P.); VarricchioC@cardiff.ac.uk (C.V.); PileggiE@cardiff.ac.uk (E.P.); 2 Institute of Pathophysiology and Allergy Research, Medical University of Vienna, 1090 Vienna, Austria; martin.schepelmann@meduniwien.ac.at; 3 School of Optometry and Vision Sciences, Cardiff University, Cardiff CF24 4HQ, UK; carolinekhogali@hotmail.co.uk (C.K.); morgans51@cf.ac.uk (S.R.M.); ian.boostrom@gmail.com (I.B.); RozanowskaMB@cardiff.ac.uk (M.R.); 4 Cardiff Institute for Tissue Engineering and Repair (CITER), Cardiff University, Cardiff CF10 3NB, UK; 5 Swansea University Medical School, Institute of Life Sciences 2, Swansea University, Swansea SA2 8PP, UK; salvatore.ferla@swansea.ac.uk; 6 Department of Chemistry, Swansea University, Swansea SA2 8PP, UK; marcella.bassetto@swansea.ac.uk

**Keywords:** molecular modelling, structure-based virtual screening, molecular dynamics, rhodopsin, small-molecules, severe inherited eye diseases

## Abstract

Accumulation of misfolded and mistrafficked rhodopsin on the endoplasmic reticulum of photoreceptor cells has a pivotal role in the pathogenesis of retinitis pigmentosa and a subset of Leber’s congenital amaurosis. One potential strategy to reduce rhodopsin misfolding and aggregation in these conditions is to use opsin-binding compounds as chemical chaperones for opsin. Such molecules have previously shown the ability to aid rhodopsin folding and proper trafficking to the outer cell membranes of photoreceptors. As means to identify novel chemical chaperones for rhodopsin, a structure-based virtual screening of commercially available drug-like compounds (300,000) was performed on the main binding site of the visual pigment chromophore, the 11-*cis*-retinal. The best 24 virtual hits were examined for their ability to compete for the chromophore-binding site of opsin. Among these, four small molecules demonstrated the ability to reduce the rate constant for the formation of the 9-*cis*-retinal-rhodopsin complex, while five molecules surprisingly enhanced the formation of this complex. Compound **7**, **13**, **20** and **23** showed a weak but detectable increase in the trafficking of the P23H mutant, widely used as a model for both retinitis pigmentosa and Leber’s congenital amaurosis, from the ER to the cell membrane. The compounds did not show any relevant cytotoxicity in two different human cell lines, with the only exception of **13**. Based on the structures of these active compounds, a series of in silico studies gave important insights on the potential structural features required for a molecule to act either as chemical chaperone or as stabiliser of the 11-*cis*-retinal-rhodopsin complex. Thus, this study revealed a series of small molecules that represent a solid foundation for the future development of novel therapeutics against these severe inherited blinding diseases.

## 1. Introduction

In the healthy retina, light absorption triggers photoisomerisation of the visual pigment chromophore 11-*cis*-retinal, covalently bound to opsin—forming rhodopsin—thus initiating the phototransduction cascade. Photoisomerised all-*trans*-retinal is then released from opsin and recycled into its 11-*cis* isomer through a series of enzymatic reactions called the visual cycle [1]. This process is dependent upon rhodopsin folding and localisation from the endoplasmic reticulum (ER) to the membranes, which are then incorporated into photoreceptor outer segments, where it acts as G-protein coupled receptor and activates the G-protein transducin upon absorption of light [2]. Different inherited blinding diseases are caused by opsin mutations, which impair its proper folding (retinitis pigmentosa, RP), or by an inability to synthesise 11-*cis*-retinal (Leber’s congenital amaurosis, LCA), which acts as a chemical chaperone for the protein and enables its folding and localisation to the outer segment photoreceptor membrane.

**Figure 1:**
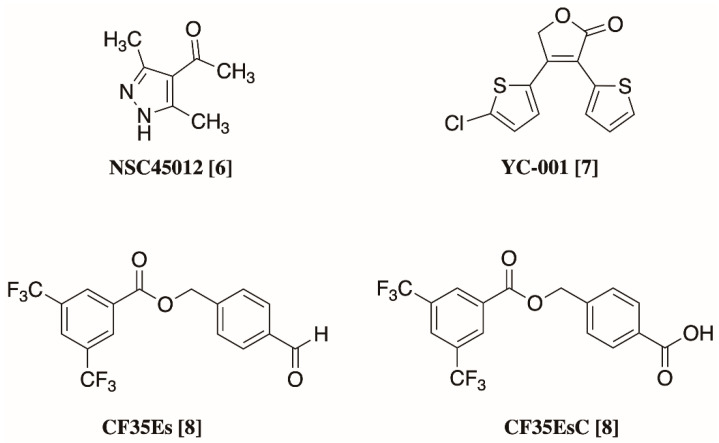
Chemical structures of non-retinoid small molecules previously reported for their ability to act as pharmacological chaperones for opsin [6,7,8].

RP and LCA are severe blinding diseases, which lead to irreversible progressive vision impairment and ultimately blindness. RP is the most common congenital eye dystrophy and affects 1 in 4000 people; in 40% of cases of RP structurally destabilising mutations in the rhodopsin gene (RHO), such as P23H, prevent the correct folding and trafficking of rhodopsin to the membrane [3]. LCA instead is the most severe retinal dystrophy in infants: it causes blindness before one year of age, and in 30% cases it is due to an inability to synthesise the visual pigment chromophore 11-*cis*-retinal, whose absence also leads to opsin misfolding and mistrafficking [4]. In these conditions, unfolded opsin molecules aggregate and accumulate in the ER, inducing ER stress and causing the death of photoreceptors, leading to progressive vision loss [5]. A cure is currently not available for these protein conformational diseases, and even if potential treatments such as gene therapy and 9- or 11-*cis*-retinal precursors are under investigation, none of them can slow down the progression of retinal degeneration and the loss of photoreceptive neurons [3]. One promising approach to prevent opsin misfolding and to induce it proper trafficking to the outer photoreceptor cell membrane are chemical chaperones: small-molecule compounds able to bind opsin by occupation of the main 11-*cis*-retinal binding site, thus inducing its proper folding and correct trafficking [6]. Over recent years, different compounds on top of retinoid analogues have been explored for their potential to bind wild-type and mutated opsins, and their ability to induce their physiological trafficking and interfere with ER stress and photoreceptor cell death. Figure 1 summarises the chemical structures of the most active non-retinoid structures reported so far [6,7,8].

The aim of this study was to apply computer-aided methods to identify novel small molecules able to bind opsin and eventually act as chemical chaperones to induce opsin folding and enable its proper trafficking in the absence of endogenous chromophore or in the presence of destabilising mutations. Such compounds may ultimately lead to the development of novel treatments to prevent photoreceptor death and vision loss in patients affected by RP and LCA. To attain this goal, a structure-based virtual screening of commercial compounds was performed on the main chromophore-binding site of rhodopsin, leading to the identification of different agents able to affect the rate constant (K) formation of the 9-*cis*-retinal-rhodopsin complex. Some of these molecules were able to increase the trafficking of P23H rhodopsin mutant from the ER to the cell membrane. A series of additional computational studies elucidated key structural features that support two possible mechanisms by which chemical chaperones can increase opsin structurally stability, both potentially relevant for the development of novel therapeutic options for RP and LCA.

## 2. Results and Discussion

### 2.1. Structure-Based Virtual Screening

**Figure 2:**
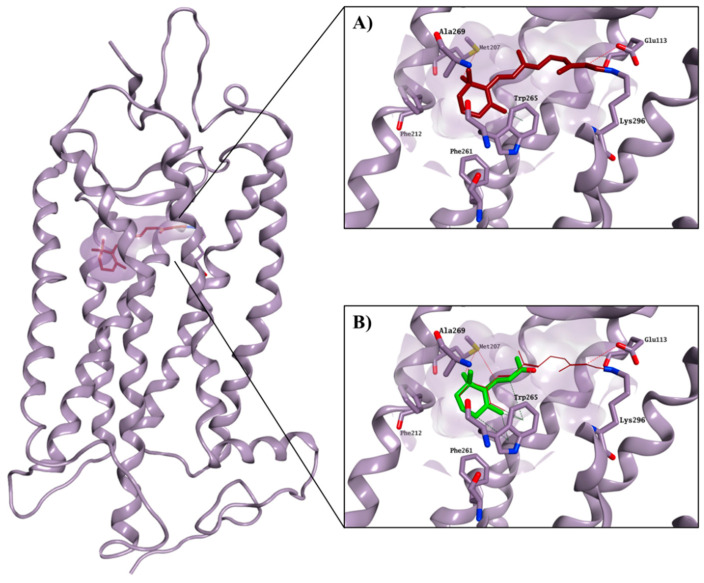
Crystal structure of bovine rhodopsin in complex with cocrystallised 11-*cis*-retinal (carbon atoms in garnet; (**A**)), and with predicted binding mode for β-ionone (carbon atoms in green; (**B**)). The binding site area is represented as the molecular surface. Rhodopsin is represented as the lilac ribbon. The ribbon for residues 261–271 and 289–295 is hidden for clarity.

The crystal structure of bovine rhodopsin cocrystallised with 11-*cis*-retinal (PDB ID: 1U19, 93% identity with the human rhodopsin) was used to perform a structure-based virtual screening of the SPECS library [9,10], a collection of 300,000 commercially available compounds with favourable drug-like properties. 11-*cis*-retinal is bound to the chromophore active site by forming a Schiff base with Lys296 and an H-bond with Glu113. Interactions between the ionone ring and the hydrophobic portion of the binding site formed by Met207, Phe212, Phe261, Trp265 and Ala269 further stabilise the 11-*cis*-retinal binding. The hydrophobic area is believed to be an essential recognition site for the ionone ring and for the binding to the chromophore pocket, as also confirmed by the ability of the shortened retinal derivative, β-ionone, to competitively inhibit the binding of 11-*cis*-retinal to rhodopsin, but without causing any physiological effect on opsin trafficking (Figure 2) [11,12,13]. 

**Figure 3:**
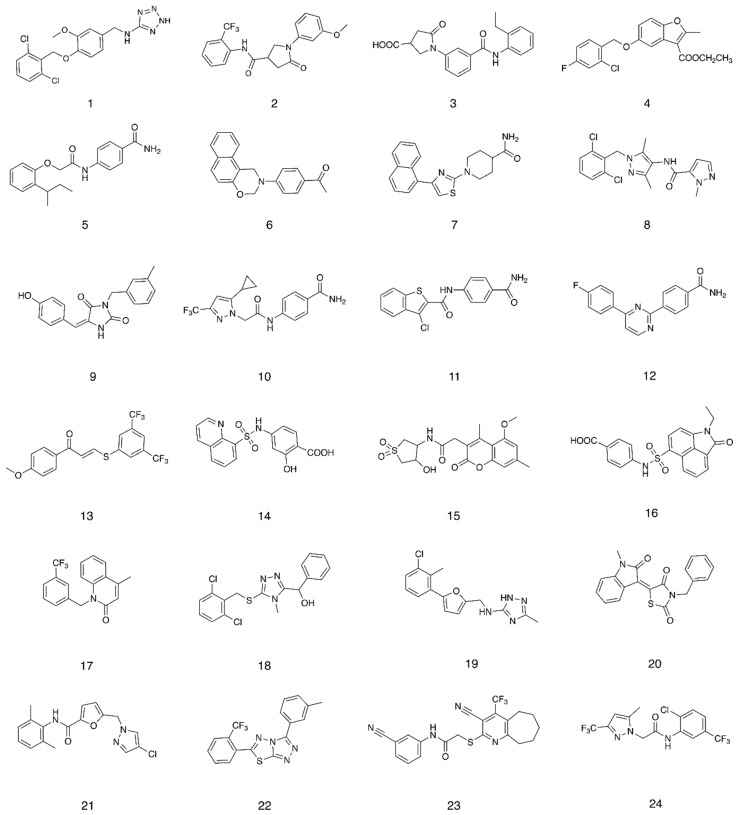
Compounds selected after the structure-based virtual screening and purchased from SPECS.

**Figure 4:**
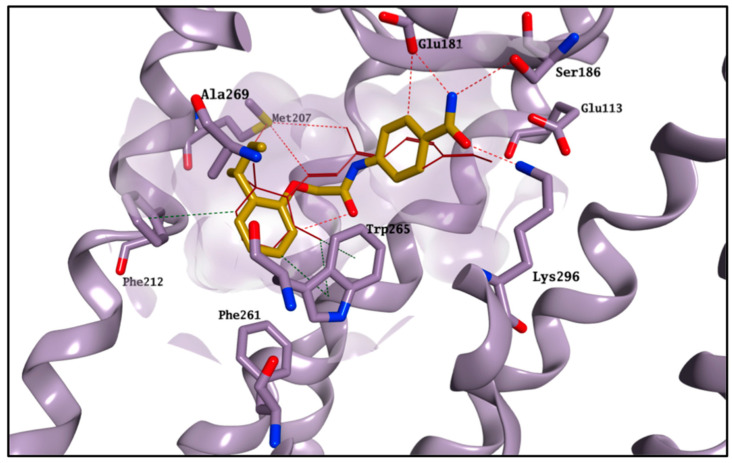
Best binding pose found for compound **5** (carbon atoms in gold). The binding site area is represented as molecular surface. Rhodopsin is represented as lilac ribbon. Ribbon for residues 261–271 and 289–295 is hidden for clarity. Cocrystallised 11-*cis*-retinal is shown in garnet.

The glide high-throughput virtual screening tool (HTVS) [14], which uses the Glide-HTVS scoring function, was employed to virtually screen the SPECS database against the selected binding area. The best 24 compounds according to this initial screening were then redocked in the 11-*cis*-retinal binding site using the more accurate standard-precision glide docking mode (GlideScore SP). In order to avoid any potential bias associated with the use of a single docking program/scoring function, the docking results (docking poses) were then rescored using three different scoring functions: Glide XP, CHEMPLP (PLANTS) and FlexX Score (Seesar) [14,15,16]. After applying a *consensus score* procedure (see Section 4.1.1) 1200 molecules were chosen and their potential interactions with the protein binding site were visually inspected. Twenty-four virtual hits were selected (Figure 3), purchased and evaluated in a competitive-binding assay (see Section 2.2). As an example, the potential binding for compound **5** is shown in Figure 4. The molecule perfectly overlaps with the cocrystallised 11-*cis*-retinal, placing its *sec*-butylbenzene portion in the hydrophobic area and forming H-bonds with Glu181, Ser186 and Lys296. The occupation of the chromophore pocket could reflect on the ability of **5** to act as potential chaperone for rhodopsin folding in the absence of its endogenous ligand.

### 2.2. Competitive Binding Assay

**Figure 5:**
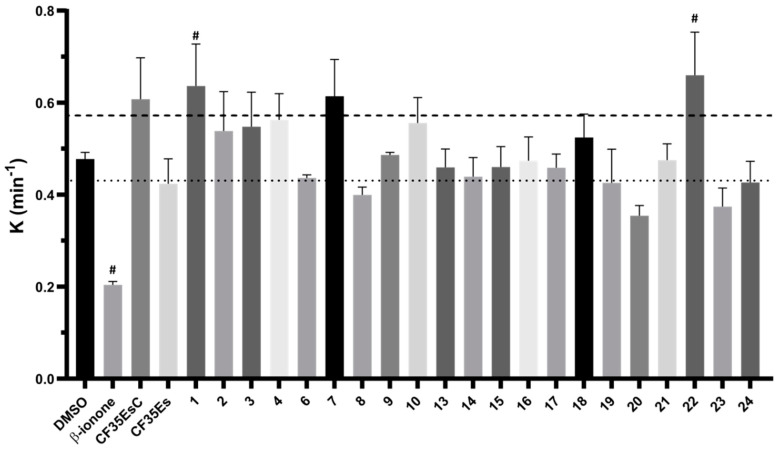
Effect of compounds **1**–**24** (compound concentration 10 times 9-*cis*-retinal) on the rate constant (K) of bovine isorhodopsin regeneration. After bleaching, compounds were preincubated for 30 min followed by addition of 9-*cis*-retinal. **β-ionone**, **CF35EsC** and **CF35Es** were used as positive controls. Compounds were considered ‘hit’ when K ≥ 10% decreased (dotted line) or ≥20% increased (dashed line) compared to DMSO. Bars represent mean ± SEM of pooled data (at least three independent measurements). Data were analysed using one-way ANOVA, followed by Fisher’s LSD (Least Significant Difference) test versus DMSO controlled for false discovery rate (FDR) by the two-stage step-up method of Benjamini, Krieger and Yekutieli # = discovery with *q* < 0.05. No data were obtained for **5**, **11** and **12** due to solubility issues at the tested concentration.

The 24 compounds selected in silico were evaluated for their ability to bind rhodopsin, by monitoring the regeneration rate of bovine isorhodopsin through time-dependent UV–Vis spectroscopy. As previously reported, the addition of 9-*cis*-retinal to rhodopsin, a chromophore with similar photoactivation properties but more stable than 11-*cis*-retinal, resulted in a time-dependent increase in optical density at 485 nm [1,17]. A compound able to compete with 9-*cis*-retinal for the binding to rhodopsin should reduce the rate constant (K) of the rhodopsin-9-*cis*-retinal complex formation. The compounds, tested at fixed concentration (Section 4.2.2), were preincubated for 30 min with freshly bleached isorhodopsin followed by addition of 9-*cis*-retinal. The rate constant K of the complex regeneration was calculated and compared to mock preincubated samples with vehicle only (DMSO, K = 0.48 ± 0.01 min^−1^, t_1/2_ = 1.52 ± 0.1 min, mean ± SEM). **β-ionone**, **CF35EsC** and **CF35Es** were used as references. **CF35EsC** and **CF35Es** were synthesised as reported in the (Supplementary Materials Figure S1). As expected, β-ionone significantly reduced the regeneration kinetic (K = 0.20 ± 0.01 min^−1^, t_1/2_ = 3.52 ± 0.3 min), confirming its ability to occupy the chromophore binding site (Figure 5 and Table S1). 

Both standards, **CF35EsC** and **CF35Es**, have been previously reported as being able to induce proper trafficking of P23H rhodopsin from ER to the cell surface, but their direct binding to the chromophore active site has only been speculated and not directly confirmed [8]. Our data suggest that while **CF35Es**, to some extent, competed for the occupation of the chromophore site, as shown by the 10% decrease of K value (K = 0.42 ± 0.05 min^−1^) compared to DMSO, **CF35EsC** possessed an unexpected ability to increase the K value by over 20% (K = 0.61 ± 0.09 min^−1^). Combining our data with what was previously reported [8], **CF35EsC** could act as an allosteric modulator of rhodopsin, either by facilitating the retinal access to the binding site enhancing and speeding up the formation of the opsin-9-*cis*-retinal complex, or by stabilising the already formed complex. Previous studies have reported β-ionone as an enhancer of the catalytic activity of different visual pigments, in which the chromophore-binding site was already occupied, potentially acting as an allosteric modulator [18]. Ortega et al. described a series of flavonoids as potential allosteric modulators able to enhance opsin stability by modulating its conformation [19]. In a similar way, **CF35EsC** could still occupy the binding site of retinal, but the ability of facilitating/stabilising the rhodopsin-9-*cis*-retinal complex formation appears to be predominant. In general, compounds able to stabilise rhodopsin (stabilisers) and prevent its degradation, in the absence of 11-*cis*-retinal (LCA) or in the presence of aberrant mutations that impair its folding and structural stability (RP), could provide a novel therapeutic mechanism to further explore, in addition to the desired chaperone function by occupation of the main chromophore binding site. Of the 24 molecules tested, four compounds (**6**: K = 0.43 ± 0.01 min^−1^; **8**: K = 0.40 ± 0.02 min^−1^; **20**: K = 0.35 ± 0.02 min^−1^; **23**: K = 0.37 ± 0.04 min^−1^) exhibited a promising decrease by 10% (or over) of the rate constant K, showing the ability to bind the chromophore pocket and compete with 9-*cis*-retinal. In particular, **20** presented the best activity profile reducing K by 20%. Interestingly, five molecules (**1**: K = 0.64 ± 0.09 min^−1^; **4**: K = 0.56 ± 0.06 min^−1^; **7**: K = 0.61 ± 0.08 min^−1^; **10**: K = 0.56 ± 0.06 min^−1^; **22**: K = 0.66 ± 0.09 min^−1^) presented the same behaviour found for **CF35EsC** increasing the K value by 10–20%, potentially possessing the ability to stabilise and enhance the opsin-9-*cis*-retinal complex formation.

No pan-assay interference compounds (PAINS) were found on the molecules presenting activity after checking their chemical structure in two different web servers [20,21], providing at this stage two potential different classes of compounds with therapeutic potential for RP and LCA: molecules, which compete for the binding to the chromophore pocket, which could act as chemical chaperones, and molecules, which seem able to facilitate/stabilise the opsin-9-*cis*-retinal complex, which could act as opsin stabilisers by binding to a different site. Both types of molecules were further explored both in silico and in vitro, as detailed below.

### 2.3. Molecular Modelling Studies on the Chromophore Binding Pocket

In order to further explore the binding of our hit molecules to opsin, 100 ns molecular dynamic (MD) simulations were performed on the rhodopsin structure, both free and in complex with the cocrystallised 11-*cis*-retinal, using the Desmond software package [22,23]. All simulations were run in triplicate. Overall, after an initial 40 ns of equilibration, the presence of 11-*cis*-retinal seemed to confer a higher stability to rhodopsin, with the simulation system converging around a fixed RMSD value, as shown by the small C-alpha RMSD variation (Figure S2). On the contrary, the ligand-free opsin was not able to reach stability after 40 ns and the RMSD value was still growing toward the end of the simulation (Figure S2). Interestingly, this result seemed in line with the findings that 11-*cis*-retinal is required to enhance rhodopsin intrinsic stability and it is an indication of the reliability of the simulation system used [1,17,19]. In order to validate the binding mode suggested by the docking program, and to find a rational discrimination between active and inactive molecules, a series of 100 ns MD simulations were also carried out on selected compounds (**6**, **8**, **9**, **13**, **17**, **20**, **21**, **22**, **23**, **CF35EsC** and **CF35Es**). The compounds’ relative binding free energies (ΔG_binding_) were then calculated using the Prime/MM-GBSA calculation method (Table 1) [24].

**Figure 6:**
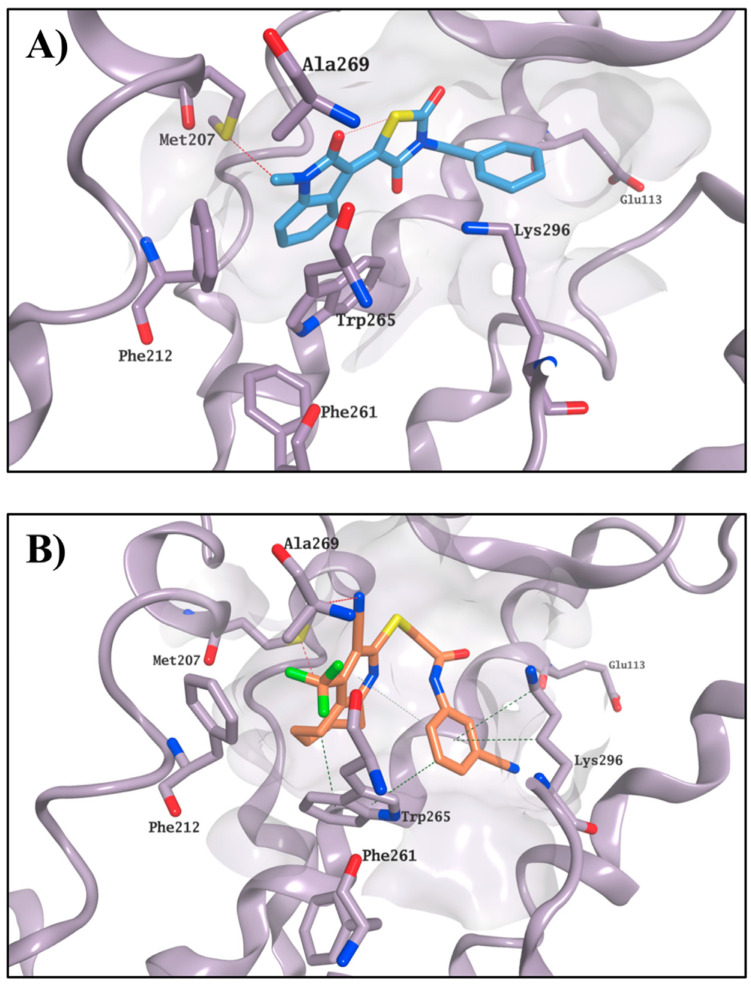
Binding mode for **20** (carbon atoms in cyan) (**A**) and **23** (carbon atoms in orange) (**B**) after MD simulation. The binding site area is represented as the molecular surface. Rhodopsin is represented as the lilac ribbon. Ribbon for residues 261–271 and 289–295 is hidden for clarity.

All the protein–ligand systems, with the exception of the ones with compounds **9**, **21** and **22**, reached stability after 40 ns, in line with the 11-*cis*-retinal-rhodospin complex, and therefore only the remaining 60 ns of the simulations were considered in our analysis (Figures S3 and S4). In general, the four active molecules (**6**, **8**, **20** and **23**) tended to optimise their occupation of the active site, maintaining a stable position during the entire MD simulation. In particular, the compounds seemed to adjust their orientation toward the hydrophobic portion of the binding site, creating hydrophobic contacts with the surrounding residues (i.e., Met207, Phe212 and Trp265), which were maintained for the entire simulation. The stable occupation of this area could confer to these four molecules their ability to compete for the chromophore-binding pocket. Moreover, the ΔG_binding_ values obtained further confirmed their potential to interact with the active site and appeared to be in line with the competitive bidding assay results. The best ΔG_binding_ was found for compound **20**, in line with this compound’s lowest observed K value. Binding of **20** and **23** is shown in Figure 6 as an example. 

**Figure 7:**
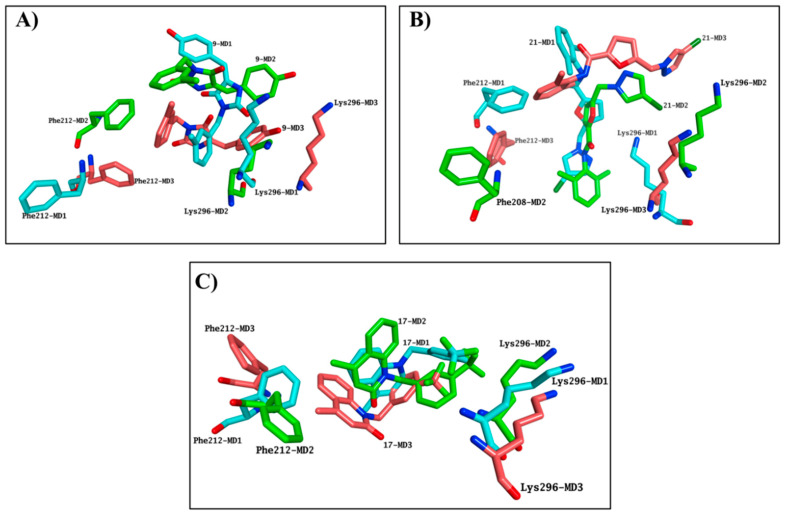
Compound **9** (**A**), **21** (**B**) and **17** (**C**) provided highly variable results in terms of occupation of the binding site, exhibiting a substantial different binding mode in each MD simulation performed. The last frames from each MD triplicate have been superposed. Only Phe212 and Lys296 are shown and the rest of rhodopsin is hidden for clarity. Carbon atoms colours: MD1-cyan MD2-green, MD3-salmon.

Simulation systems for **9** and **21** were not able to equilibrate during the entire MD, indicating that these molecules were not likely to bind the chromophore pocket. Both molecules presented highly variable results in terms of occupation of the binding site in each simulation performed, further confirming the inability of **9** and **21** to consistently occupy the chromophore pocket, in line with the negative results obtained in the competitive binding assay (Figure 7A,B). Although the **17**-rhodhopsin complex does reach stability after 40 ns, as with the active molecules, variable binding modes were obtained, indicating that **17** is also not likely to compete for binding to the active site (Figure 7C). 

**Figure 8:**
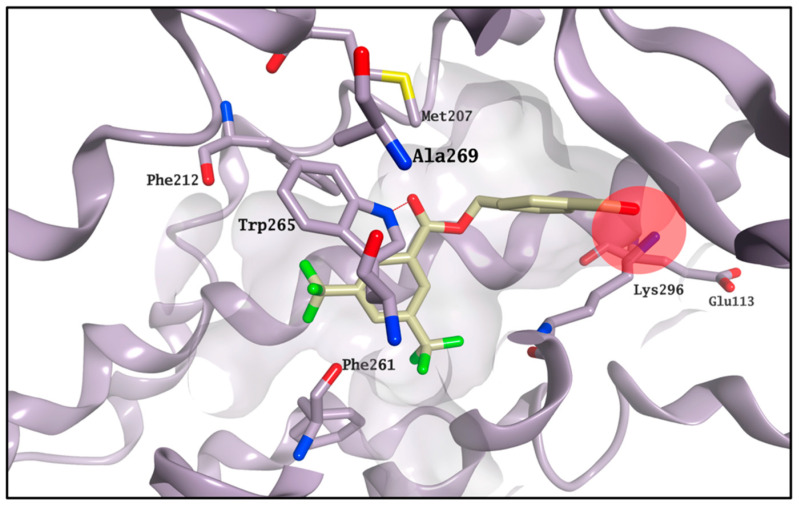
Binding mode for **CF35Es** (carbon atoms in light yellow) after MD simulation. The aldehyde moiety of **CF35Es** is placed nearby Lys296 during the entire simulation. The binding site area is represented as the molecular surface. Rhodopsin is represented as the lilac ribbon. Ribbon for residues 261–271 and 289–295 is hidden for clarity.

The MD results predict that **13** should bind to the chromophore active site, with its ΔG_binding_ suggesting it should provide a reduction of the K value similar to **6**. This inconsistency between the competitive binding assay and the molecular modelling prediction could be caused by the simultaneous presence of both potential effects detected on the competitive binding assay, which could affect the final read out of the assay itself. **13** may still occupy the main chromophore binding area, as predicted by the MD simulations, but it may also be able to stabilise the formation of the rhodopsin-9-*cis*-retinal complex, thus affecting the outcome of the competitive binding assay. The failure of the simulation system to reach equilibration in all the three experiments for **22**, associated with the highest increase for the K value, suggests that this compound is not likely to compete for the chromophore active site, and it may act purely as a stabiliser of the rhodopsin-9-*cis*-retinal complex. According to the calculated ΔG_binding_, carboxylic acid derivative **CF35EsC** seems to possess a limited ability to bind the chromophore site, in line with the competitive binding assay results. On the other hand, the aldehyde moiety on **CF35Es** could form a transient Schiff base with rhodopsin (ΔG_binding_ calculation cannot predict covalent interactions), as also confirmed by the vicinity of this group to Lys296 during the MD simulation (Figure 8), potentially making this compound much more stable in the binding site than **CF35EsC,** thus potentially explaining its ability to reduce the K value.

**Figure 9:**
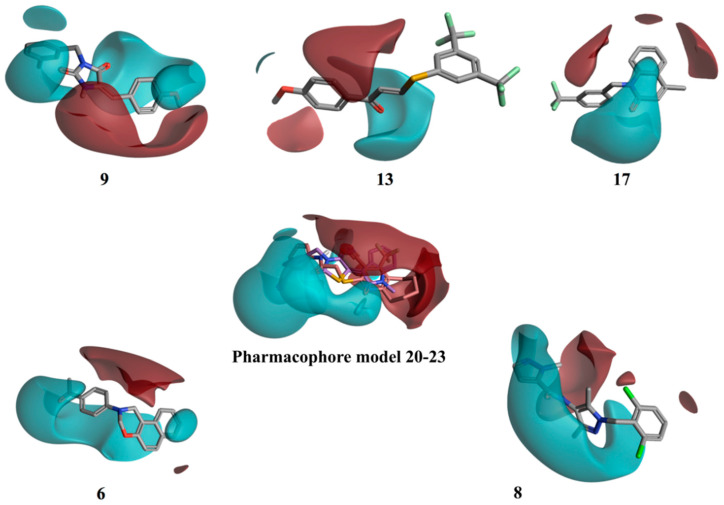
3D electrostatic–hydrophobic and shape properties. The pharmacophore model generated from **20** and **23** presents two well distinct and separated regions: a positive electrostatic region, in red, and a negative electrostatic region, in cyan. The rest of the compounds were aligned with the pharmacophore, using a field-based alignment approach. Results for **6** and **8** are shown as an example of compounds matching the pharmacophore.

In order to find possible common structural features among the compounds able to bind the chromophore pocket, a pure ligand-based approach was performed. This approach uses a molecular field points-based similarity method to generate a series of low-energy conformations for each compound [25,26]. The molecular field points define the shape, electrostatic and hydrophobic properties of a molecule and their spatial distribution. The lowest energy conformation and its associated 3D electrostatic-hydrophobic and shape properties for **20** and **23**, the compounds showing the best activity profile, were then generated. These electrostatic and hydrophobic properties of the two conformations obtained were then compared and used to derive a pharmacophore model to identify common motifs between the two active molecules [26,27]. Figure 9 shows the resulting pharmacophore model. Two well distinct and separated regions can be identified: a positive electrostatic region, in red, and a negative electrostatic region, in cyan. The other compounds were then aligned with the identified pharmacophore, using a field-based alignment approach, to identify potential differences between active and inactive molecules. Interestingly, the best alignment results obtained for the inactive/weakly active compounds present a quite different distribution of the two electrostatic regions in comparison with the active pharmacophore query. The separation between the positive and the negative region was less distinct, with the distribution of the negative filed area remarkably less wide (**13** and **17**), or in a completely different orientation (**9**), for the inactive compounds (Figure 9). Compounds **6** and **8** are characterised by a wide negative electrostatic region oriented correctly, matching the pharmacophore model for the active molecules (Figure 9).

**Figure 10:**
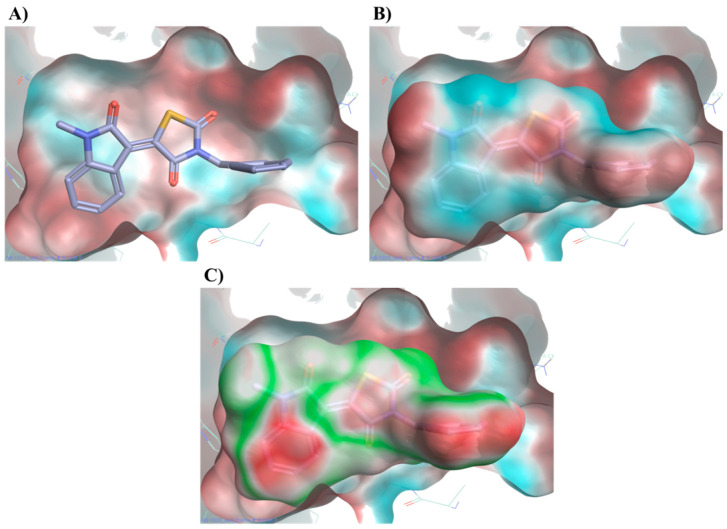
Electrostatic potential (ESP) of the binding pocket (**A**) and of compound **20** (**B**). Red colour indicates a positive ESP region and blue colour negative ESP region. **20** places its large negative electrostatic area (blue) in correspondence of the positive portion of the protein (red). Protein compound **20** electrostatic complementarity (**C**). Green colour indicates complementarity between protein and ligand, while red indicates an electrostatic clash. Binding mode for compound **20** obtained after MD simulation.

A further calculation of the protein–ligand electrostatic complementarity for some of the active molecules, comparing the protein and ligand electrostatic potential (ESP) values, revealed the presence of a positive ESP surface on the binding pocket that could facilitate the binding of a ligand possessing a large negative ESP [26,28,29]. Figure 10 displays compound **20** placing its large negative electrostatic area, having as the centre the thiazolidinedione ring, in correspondence to this positive portion of the protein, showing a high electrostatic complementary with the binding pocket. According to these findings, the presence of a well-defined negative electrostatic region on the compound appears to be an essential feature, and its electrostatic complementarity with the positive portion of binding site a critical factor, for the ligand–protein interaction.

### 2.4. Molecular Modelling Studies to Investigate the Observed Stabilisation Effect of the Rhodopsin-9-cis-Retinal Complex

**Figure 11:**
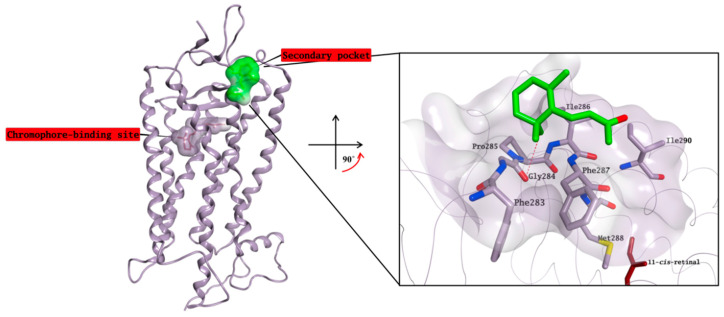
Crystal structure of β-ionone (carbon atoms in green) in complex with rhodopsin in which the chromophore-binding pocket is already occupied by 11-*cis*-retinal (carbon atoms in garnet). β-ionone is bound to a small, surface-exposed and highly hydrophobic pocket. The binding site area is represented as the molecular surface. Rhodopsin is represented as the lilac ribbon. On the binding site cut out rhodopsin is represented as a lilac tube for clarity.

According to our competitive binding assay, some of the selected molecules increase the rate constant K for the formation of the rhodopsin-9-*cis*-retinal complex, and could therefore act as allosteric modulators/stabilisers of rhodopsin structure, either by facilitating the retinal access to its main binding site, or by stabilising its interaction with this site. Different alternative binding pockets on the opsin structure have been previously suggested, but none of them has been directly confirmed through cocrystallisation with any active molecule so far [19,30]. Although the exact mechanism behind the ability of β-ionone to increase the catalytic activity of different visual pigments without interacting with the chromophore-binding site is still not known [18], a crystal structure in complex with rhodopsin in which the chromophore-binding pocket is already occupied by 11-cis-retinal has been resolved by Makino et al. [13]. In this structure, β-ionone is bound to a small, surface-exposed and highly hydrophobic pocket formed by Phe283, Gly284, Pro285, Ile286, Phe287, Met288 and Ile290, mainly forming hydrophobic interactions with the surrounding residues (Figure 11). The pocket is not distant from the primary chromophore-binding site, and it is present in both bovine opsin-11-cis-retinal complex and in ligand-free opsin crystal structures [9,31]. Furthermore, Behnen et al., studied the effect of different mutations in weakening the network of native links that confers stability to opsin, and some of these mutations weaken the network of interactions mainly in an area that is in close proximity to the β-ionone binding pocket [32]. Binding of β-ionone or other small molecules in this pocket could have a stabilising effect on this network, enhancing the intrinsic stability of native opsin.

**Figure 12:**
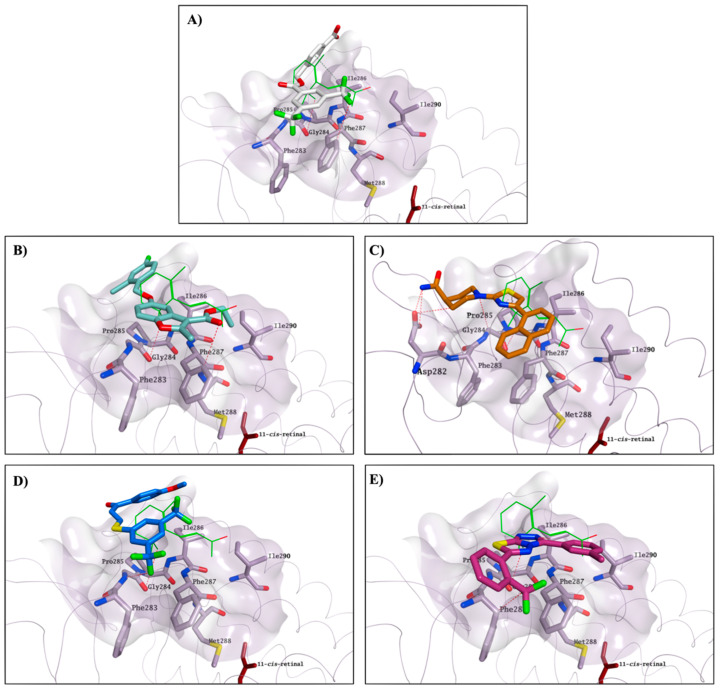
Potential binding mode for **CF35EsC** (carbon atoms in white; (**A**)), **4** (carbon atoms in teal; (**B**)), **7** (carbon atoms in orange; (**C**)), **13** (carbon atoms in blue; (**D**)) and **22** (carbon atoms in red violet; (**E**)) to the secondary hydrophobic pocket. β-Ionone carbon atoms are in green, whereas 11-*cis*-retinal carbon atoms are garnet. The binding site area is represented as the molecular surface. Rhodopsin is represented as a lilac tube for clarity.

The identified compounds able to increase K for the formation of the opsin-9-*cis*-retinal complex could likely interact with the same secondary pocket, thus facilitating/stabilising the formation of the opsin-11-*cis*-retinal complex. Molecular docking studies show that all compounds able to increase K (**1**, **4**, **7**, **10**, **13** and **22**)**,** on top of the two reference molecules included in our assay (**CF35EsC** and **CF35Es**), have the potential to interact with this secondary pocket, placing one hydrophobic portion of their structure inside the binding cavity, similarly to the binding of the β-ionone cyclohexenyl ring found in the crystal structure. Predicted binding of **CF35EsC**, **4**, **7**, **13** and **22** to this secondary site is shown in Figure 12. Compound **7** is also establishing multiple H-bonds with Asp282, which can further stabilise the molecule on the binding site.

**Figure 13:**
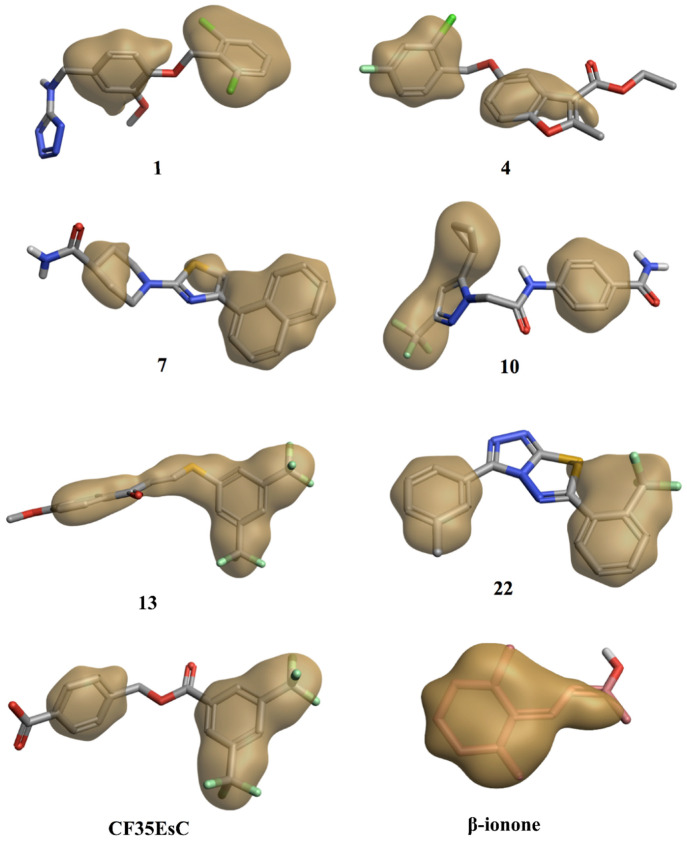
Hydrophobic region of the potential allosteric compounds. Compounds present either an extended planar hydrophobic region or two hydrophobic areas with a spatial orientation, which is relatively coplanar. The best scored molecular docking pose for each compound on the secondary binding pocket and the cocrystallised β-ionone were used for the hydrophobic surface calculation.

**Figure 14:**
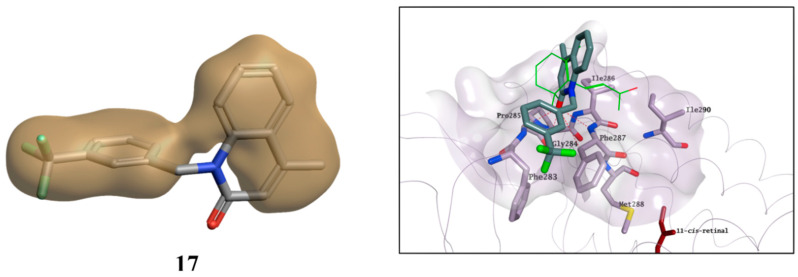
Hydrophobic region for **17** and its potential binding mode on the secondary pocket. The compound presents two hydrophobic areas, which are not coplanar. β-ionone carbon atoms are in green. The binding site area is represented as molecular surface. Rhodopsin is represented as the lilac tube for clarity.

Interestingly, the chemical structure of all these compounds is characterised by either an extended planar hydrophobic region, or by two hydrophobic areas with a spatial orientation, which is relatively coplanar (Figure 13). Combination of hydrophobicity and coplanarity may be an essential feature to correctly occupy this shallow hydrophobic pocket, and to act as potential allosteric modulators of rhodopsin. Although **17** is characterised by two hydrophobic areas, the lack of coplanarity between them does not allow the molecule to correctly interact with the pocket, in line with its inability to affect K found in the competitive binding assay (Figure 14).

### 2.5. Evaluation of Cytotoxic Effects for Selected Hit Compounds

**Figure 15:**
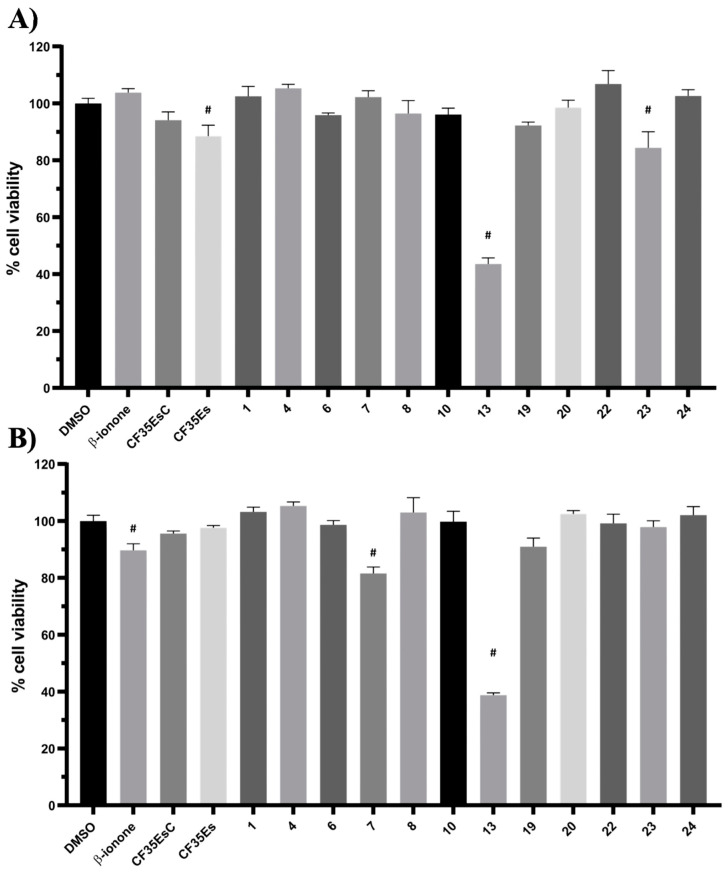
Potential cytotoxic effect for selected compounds on HepG2 (**A**) and ARPE-19 (**B**) at 25 µM. The cell viability was determined as a percentage of vehicle control-treated cells (DMSO). Bars represent mean ± SEM of at least three independent measurements. Data were analysed using a one-way ANOVA, followed by a Fisher’s LSD test versus DMSO controlled for false discovery rate (FDR) by the two-stage step-up method of Benjamini, Krieger and Yekutieli. # = discovery with *q* < 0.05.

The most interesting compounds, including the two standards and β-ionone, were tested for their potential cytotoxicity using two different cell lines: HepG2 and ARPE-19. HepG2 cells are widely used as an in vitro model for the detection of liver toxicity [33], while ARPE-19 cells are models for human retinal pigment epithelial cells. Most compounds showed no toxic effect in both cell lines (Figure 15), with the only exception of **7** and **23**, which reduced ARPE-19 and HepG2cell viability by around 20% respectively, and **13**, which reduced cell viability by around 60% in both cell lines. Overall, the compounds can be considered as not having any relevant cytotoxic effect, except for **13**, and were then evaluated for their pharmacologic potential in a more specific rhodopsin rescue cell-based assay.

### 2.6. Immunofluorescence Microscopy

**Figure 16:**
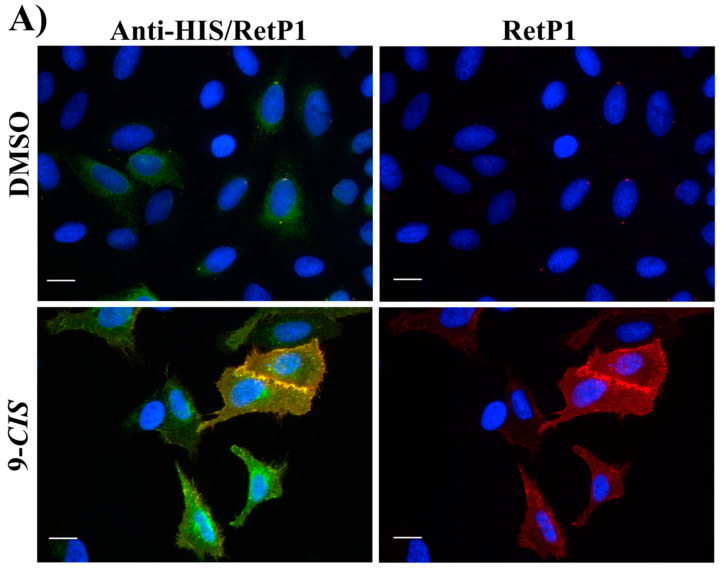

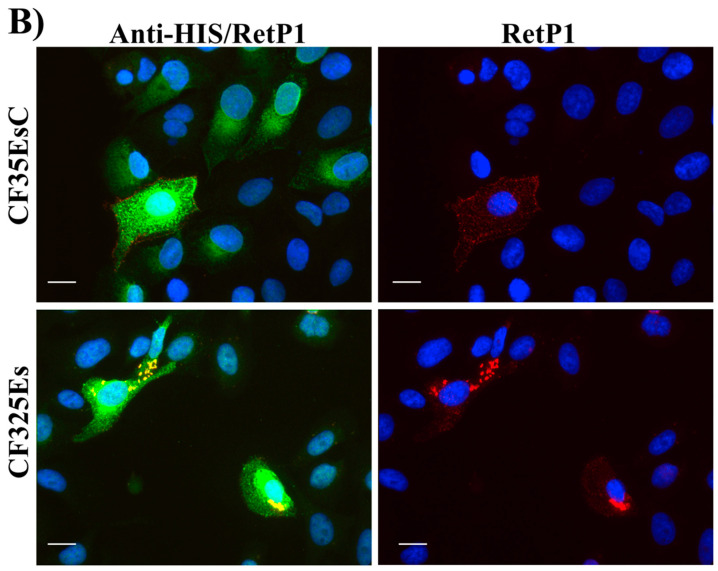
Subcellular localisation of hRHO P23H His-Tag in the presence of 9-cis-retinal (**A**), **CF35EsC** and **CF35Es** (**B**). 9-cis-retinal was tested at 5 µM, whereas **CF35EsC** and **CF35Es** at 20 µM. Anti-His/RetP1: merged image of RetP1 staining (red) and Anti-His Tag binding total rhodopsin staining (His Tag expressed at the C-terminus of rhodopsin, intracellular, post-permeabilisation with 0.1% Triton; green). RetP1: anti-rhodopsin antibody (extracellular N-terminus antibody, incubation prior to permeabilisation, red) staining only the protein exposed on the cell membrane. Nuclei were stained with DAPI (blue). Scale bars: 10 μm.

**Figure 17:**
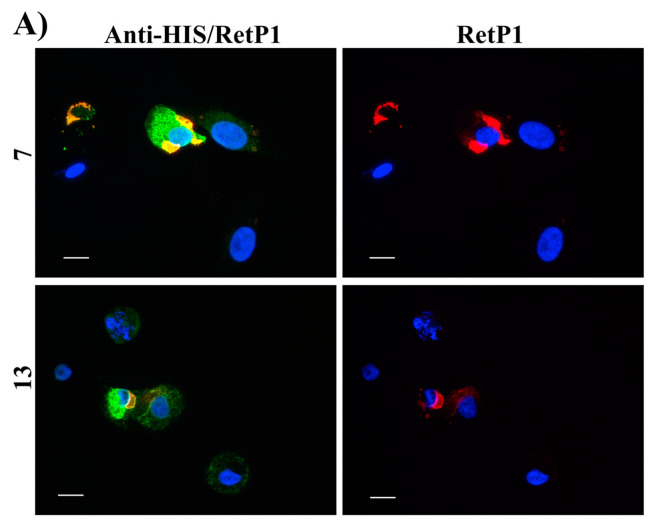

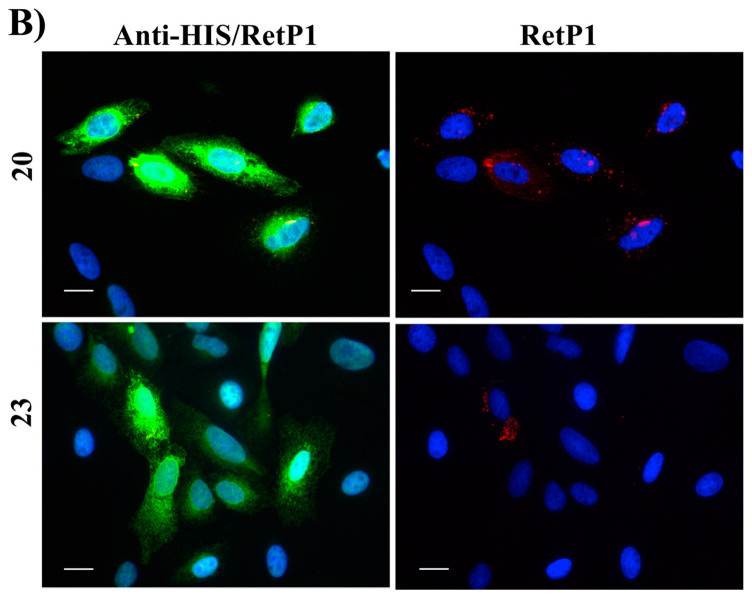
Subcellular localisation of hRHO P23H His-Tag in the presence of tested compounds: **7** and **13** (**A**); **20** and **23** (**B**). **13** and **20** were tested at 10 µM, whereas **7** and **23** at 20 µM. Anti-His/RetP1: Merged image of RetP1 staining (red) and Anti-His Tag binding total rhodopsin staining (His Tag expressed at the C-terminus of rhodopsin, intracellular, post-permeabilisation with 0.1% Triton; green). RetP1: Anti-rhodopsin antibody (extracellular N-terminus antibody, incubation prior permeabilisation, red) staining only the protein exposed on the cell membrane. Nuclei were stained with DAPI (blue). Scale bars: 10 μm.

**Figure 18:**
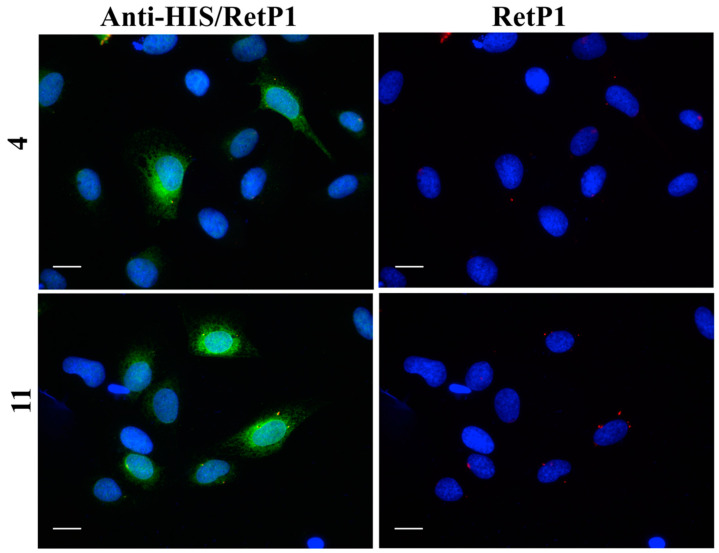
Subcellular localisation of hRHO P23H His-Tag in the presence of tested compounds (representative images): **4** and **11. 4** were tested at 20 µM, whereas **11** at 10 µM Anti-His/RetP1: merged image of RetP1 staining (red) and Anti-His Tag binding total rhodopsin staining (His Tag expressed at the C-terminus of rhodopsin, intracellular, post-permeabilisation with 0.1% Triton; green). RetP1: anti-rhodopsin antibody (extracellular N-terminus antibody, incubation prior permeabilisation, red) staining only the protein exposed on the cell membrane. Nuclei were stained with DAPI (blue). Scale bars: 10 μm.

In this experiment, the localisation of P23H human rhodopsin His-tag (hRHO P23H His-Tag) was evaluated by immunostaining under both cell membrane non-permeabilised and membrane-permeabilised conditions. Rhodopsin trafficked to the cell membrane was detected with an anti-rhodopsin antibody (RetP1, which recognise an extracellular epitope) under non-permeabilised conditions. The total expressed rhodopsin was then detected with an anti-His-tag antibody after the cell membrane was permeabilised. In U2OS cells transiently transfected with hRHO WT His-tag, anti-rhodopsin staining (red) showed proper trafficking of rhodopsin with homogeneous distribution on the cell membrane in the presence of 9-*cis*-retinal that was minimally affected by the absence of 9-*cis*-retinal, as expected for wild-type human rhodopsin in this assay (Figure S5). This is in line with previous findings [34] and suggests that a small tag (His-tag) at the C-terminus of wild-type rhodopsin does not affect its correct trafficking to the membrane. Additionally, in accordance with a previous study [34], in the established cell line, P23H mutant rhodopsin failed to show proper trafficking and a homogeneous cell surface distribution, which was substantially rescued when cells were incubated with 9-*cis*-retinal (Figure 16A). Cells treated with the two standards **CF35EsC** and **CF35Es** also showed a mild improvement in P23H mutant opsin localisation on the membrane (Figure 16B, red). Among our hit compounds, only **7**, **13**, **20** and **23** elicited a detectable increase in the trafficking of P23H mutant from the ER to the cell membrane (Figure 17A,B). For the remaining compounds, only reticular distribution consistent with ER retention was detected (Figure 18). According to our molecular modelling studies, **7** and **13** are likely to act as rhodopsin **stabilisers**, with a stabilisation effect of rhodopsin 3D structure by binding to a secondary site, whereas **20** and **23** are likely to act as chemical **chaperones**, competing for the binding to the main chromophore pocket. In both cases, the correct folding of mutant P23H rhodopsin was partially restored, allowing its proper trafficking to the membrane. Although **13** was found to be toxic, its mild ability of increasing the trafficking of the P23H mutant can be considered a very promising starting point for further development of its molecular scaffold.

## 3. Conclusions and Future Works

In the presented work, the chromophore-binding site of rhodopsin was selected to perform a structure-based virtual screening of commercially available, drug-like compounds (300,000). Rhodopsin is involved in the transmission of the visual signal in the retina and its misfolding/mistrafficking, due to the absence of endogenous chromophore 11-*cis*-retinal or to structurally destabilising mutations, causes different severe inherited eye diseases, ultimately leading to blindness. The development of small molecules chemical chaperones for rhodopsin provides an attractive approach to promote its proper folding and trafficking, which would allow to slow down photoreceptor cell death and vision loss in patients affected by retinitis pigmentosa and Leber congenital amaurosis. Twenty-four virtual hits selected with our in silico approach were examined for their ability to compete with 9-*cis*-retinal for the binding of rhodopsin chromophore site, monitoring the regeneration rate of bovine isorhodopsin through time-dependent UV–Vis spectroscopy. Four candidates were found to have a promising ability to reduce the rate constant (K) for the formation of the 9-*cis*-retinal-opsin complex by 10–20%, acting as potential chemical chaperones for opsin. Five molecules were found to facilitate/stabilise the opsin-9-*cis*-retinal complex by increasing the rate constant for its formation, thus revealing their potential to act as stabilisers of the protein 3D structure. Among these hits in the first assay, **7**, **13**, **20** and **23** were able to induce the correct folding of mutant P23H rhodopsin, allowing its proper trafficking to the membrane in a fluorescent immunohistochemistry cell-based assay. These compounds did not show any relevant cytotoxicity in two different human cell lines (HepG2 and ARPE-19), with the only exception of **13**.

Molecular modelling studies revealed that in order to compete for the chromophore-binding pocket, a molecule requires a **hydrophobic region** to interact with the hydrophobic area of the active site (the recognition site for the ionone ring of 11-*cis*-retinal and β-ionone), along with a **well-defined negative electrostatic region**, electrostatically complementary with the positive portion of the binding site. A secondary binding pocket, not distant from the primary chromophore-binding site and occupied by β-ionone in one rhodopsin crystal structure, was revealed by our studies as a putative target for compounds acting as potential allosteric stabilisers of the rhodopsin structure. Molecular modelling investigations showed that a combination of **hydrophobicity** and **coplanarity** are essential features for a molecule to interact with this surface-exposed and highly hydrophobic secondary pocket.

In conclusion, different novel scaffolds were found to act as potential rhodopsin chaperones or stabilisers. Their effect to induce the correct folding of mutant P23H rhodopsin in a cell-based assay was moderate. Poor cell membrane permeability of the compounds may have prevented a more pronounced effect. Future structural optimisation will be performed to improve the physical–chemical properties of these compounds, either to optimise their cell permeability, or to reduce any associated toxicity, a priority for compound **13**.

Important insights have been obtained on the structural features required for a molecule to act either as a chaperone or stabiliser through in silico studies: these will guide further structure–activity relationship optimisation efforts of the new hit molecules, and provide a valuable piece of information to perform new ligand-based virtual screenings of larger compound libraries. These findings represent a major starting point for the continued development of novel compounds that can act as rhodopsin folding chaperones/stabilisers for the treatment of severe inherited eye diseases whose molecular basis is opsin misfolding and mistrafficking. In particular, our newly developed pharmacophoric models for the two possible binding modes to opsin lay the groundwork for these future studies.

## 4. Materials and Methods

### 4.1. Molecular Modelling

All molecular modelling experiments were performed on Asus WS X299 PRO Intel^®^ i9-10980XE CPU @ 3.00GHz × 36 running Ubuntu 18.04 (graphic card: GeForce RTX 2080 Ti). Molecular Operating Environment (MOE, 2019.10, Montreal, QC, Canada) [35], Maestro (Schrödinger Release 2020-2, New York, NY, USA) [14], PLANTS [15], Seesar (version 9.2, containing FlexX, Sankt Augustin, Germany) [16] and Cresset Inc. (2020, Litlington, UK)) [25,27,28] were used as molecular modelling software. A library of commercially available compounds was downloaded from the SPECS website (www.specs.net) in the sdf format and prepared using the Maestro LigPrep tool by energy minimising the structures (OPLS_2005 force filed), generating possible ionisation states at pH 7 ± 2 (Epik), generating tautomers, generating at most 3 stereoisomers per ligand and low-energy ring conformers. All the compounds featuring chiral centres were purchased as racemic mixtures from SPECS. Up to 3 stereoisomers per chiral compound were considered for the virtual screening process and only the best performing stereoisomer per molecule has been selected for the visual inspection. Stereochemistry of the best performing stereoisomer for chiral compounds is as follows: **2** (S), **3** (R), **5** (R), **15** (S, S) and **18** (R).

#### 4.1.1. Molecular Docking 

The crystal structure of bovine rhodopsin was downloaded from the PDB (http://www.rcsb.org/; PDB code 1U19). The protein was prepared using the MOE Protein Preparation tools, the bond between 11-*cis*-retinal and Arg296 (Schiff base) was disconnected and the resulting protein–ligand complex saved in three different format: pdb (to be used for FlexX rescore), mol2 (to be used for PLANTS rescore after removing the 11-*cis*-retinal) and mae (to be used in Maestro to perform the HTVS study).

The protein in the mae format was preprocessed using the Schrödinger Protein Preparation Wizard by assigning bond orders, adding hydrogens and performing a restrained energy minimisation of the added hydrogens using the OPLS_2005 force field. A 9 Å docking grid (inner-box 10 Å and outer-box 19 Å) was prepared using as the centroid the cocrystallised 11-*cis*-retinal. An HTVS of the SPECS library was performed using Glide HTVS precision keeping the default parameters and setting 1 as the number of output poses per input ligand to include in the solution. The best 25,000 compounds were then redocked using the more accurate Glide SP precision keeping the default parameters and setting 3 as the number of output poses per input ligand to include in the solution. The docking results obtained were then rescored using Glide XP, FlexX Score and CHEMPLP (PLANTS) scoring functions. The values of the three different scoring functions for each docking pose were then analysed together (consensus score) and only the docking poses falling in the top 25% of the score value range in all the three scoring functions were selected for the final visual inspection.

The visual inspection process, conducted as last step of the structure-based virtual screening, was performed using MOE 2019.10. The docking poses of the compounds obtained from the consensus score procedure were evaluated considering the following criteria:

- Ability of a compound to overall occupy the binding site;
- Number of interactions formed between the compound and the target protein (H-bonds, pi–pi interactions, etc.);
- Coverage of different chemical scaffolds, discarding similar chemical entities.

The crystal structure of bovine rhodopsin in complex with β-ionone was downloaded from the PDB (http://www.rcsb.org/; PDB code 3OAX). The protein was preprocessed using the Schrödinger Protein Preparation Wizard as reported above and a 12 Å docking grid (inner-box 10 Å and outer-box 22 Å) was prepared using as a centroid the cocrystallised β-ionone. Docking of the virtual hit molecules and the two standards in the new potential secondary binding site was performed using Glide SP precision keeping the default parameters and setting 5 as the number of output poses to include in the solution. The docking output database was saved as the mol2 file and the docking poses visually inspected for their binding mode in MOE. The hydrophobic surface of each compound was created in Flare using the best scored molecular docking pose on the secondary binding pocket.

#### 4.1.2. Molecular Dynamics 

Molecular dynamics simulations were performed using the Desmond package for MD simulation, employing OPLS_2005 force field in the explicit solvent and the TIP3 water model. The initial coordinates for the MD simulation were taken from the best docking poses obtained for each single compound in the structure-based virtual screening. A cubic water box was used for the solvation of the system, ensuring a buffer distance of approximately 10 Å between each box side and the complex atoms. The systems were neutralised adding 7 sodium counter ions. The system was minimised and pre-equilibrated using the default relaxation routine implemented in Desmond. A 100 ns MD simulation was performed, during which the equation of motion was integrated using a 2 fs time step in the NPT ensemble, with a temperature (300 K) and pressure (1 atm) constant. All other parameters were set using the Desmond default values. Data were collected every 8.5 ps (energy) and every 33.3 ps (trajectory). Each simulation was performed in triplicate, every time using a random seed as a starting point. Visualisation of the protein–ligand complex and MD trajectory analyses were carried out using Maestro. RMSD, secondary structure and protein–ligand interactions analyses were performed using the Simulation Event Analysis tool and the Simulation Interaction Diagram of Desmond. The ΔG_binding_ values of the protein–ligand complex were calculated using the MM/GBSA method as implemented in the Prime module from Maestro using the default settings and the Maestro script termal_mmgbsa.py. Briefly, the script takes in the MD trajectory from the last 60 ns of simulation, splits it into individual frame snapshots (extracted every 0.33 ns, for a total of 181 frames), and runs each one through MMGBSA (after deleting waters and separating the ligand from the receptor). For each simulation triplicate, an average ΔG_binding_ values for the final 60 ns was calculated.

#### 4.1.3. Molecular Field-Based Similarity and Electrostatic Complementarity Studies 

The 3D electrostatic and shape properties of compounds were constructed using Forge v3.0 software (Cresset Inc., Cambridgeshire, UK). Firstly, the FieldTemplater tool, implemented in Forge software, was used to derive a pharmacophore model by comparing the electrostatic and hydrophobic property of the active compounds **20** and **23**. Consequently, each compound was subjected to a field-based alignment to the pharmacophoric template. This method is based on a 3D-shape and electrostatic potential similarity calculated by the alignment and superposition between the reference compound and the compounds in the database according to their electrostatic distribution and volume occupied. Field point-based descriptors were used to compare the electrostatic and hydrophobic distribution of each molecule. Default settings were used.

The electrostatic complementarity was calculated using Flare v4.0 (Cresset Inc., Cambridgeshire, UK). Electrostatic complementarity (EC) maps were based on a calculation of ESP value for the ligand and the protein: regions of ligand surface are coloured green if there is perfect electrostatic complementarity with the protein, while they are coloured red if there is an electrostatic clash. Default settings were used.

### 4.2. Biological Assays

#### 4.2.1. Retina Outer Segments (ROSs) Isolation from Bovine Eyes

Bovine eyes were obtained from a local abattoir and shipped on ice in light protective containers. Isolation of ROS was performed under dim red light following a modified protocol from Papermaster [36]. Briefly, an excised retina was gently homogenised in sucrose buffer (1.14 M Sucrose, 1 M NaCl, 0.1 M MgCl_2_ and 1 M Tris-acetate in H_2_O) and separated through a sucrose density gradient (0.77 M, 0.84 M and 1.14 M sucrose) through serial ultracentrifugation (at 4 °C). Isolated ROS were resuspended in phosphate buffer (PBS) and quantified for opsin and rhodopsin content with a UV–Vis spectrophotometer using the absorption coefficient ε_280 nm_ = 81,200 M^−1^ cm^−1^ and ε_500 nm_ = 40,600 M^−1^ cm^−1^, respectively.

#### 4.2.2. Competitive Binding Assay

ROS membranes containing opsin were suspended in PBS buffer containing 1% N-dodecyl-b-maltoside (DM) at a final concentration of 20–25 µM. The compounds were incubated with the resuspended opsin for 30 min at room temperature at a concentration 10 times the predetermined concentration of opsin. The mixture was then bleached with green laser pointer light for 2 min. UV–visible spectra of the samples were measured before addition of 9-*cis*-retinal (equimolar concentration of opsin) and after treatment at time points: 0, 1, 2, 3, 4, 9, 14, 19, 24 and 29 min. Compounds CF35EsC, CF35Es [8] and β-ionone were used as positive controls. The kinetics of isorhodopsin regeneration were repeated at least three times for each condition. The time course of isorhodopsin regeneration (at 485 nm) was fitted by a one-phase association equation and rate constant (K) were calculated using GraphPad Prism 7.0 (Graphpad Software, (La Jolla, CA, USA)).

#### 4.2.3. Cell Culture

Cells were maintained in DMEM (HepG2) or DMEM/F12 1:1 (ARPE-19 and U2OS), enriched with 10% foetal bovine serum (Sigma Aldrich and Gibco) and cultured under standard culture conditions (in the dark, 37 °C, 5% CO_2_ and 95% relative humidity (rH)). All media (Gibco, Renfrew, UK and Pan Biotech, Wimborne, UK) were supplemented with antibiotics (100 units/mL penicillin and 1 µg/mL streptomycin, (Gibco, Renfrew, UK) and Glutamax (Gibco) as 1% of volume in a mixed solution. U2OS cells were kindly gifted by Prof. Trevor Dale (Cardiff University) and used to generate the stable cell line expressing—in the presence of tetracycline—the human rhodopsin gene bearing P23H mutation and a 6 histidines tag (His-Tag, C-terminus). ARPE-19, human RPE cell line was purchased from ATCC (CRL-2302), while the HepG2 cell line was kindly provided by Prof. Karl Hoffmann’s lab, IBERS (Aberystwyth University).

#### 4.2.4. Determination of Cell Viability Assessed by CellTiter-Blue

To quantify the amount of live and dead cells, CellTiter-Blue Cell Viability Assay (Promega, Southampton, UK) was used as recommended by the manufacturer. Briefly, the day prior to the assay cells were seeded in 96-well plates (1 × 10^4^ per well) in culture media containing 1 g/L glucose supplemented with 1× penicillin/streptomycin, 1× Glutamax and 2% FBS. The day after, the media was replaced with fresh media (2% FBS and 1g/L glucose) containing 25 µM of tested compound. Cells were incubated at 37 °C for 24 h. After 24 h, 20 μL of CellTiter Blue reagent was added to each well (100 μL of cell culture media) and incubated for 4 h at 37 °C. Following this period, the fluorescence was measured using excitation/emission wavelengths of 560/590 nm. Data were normalised to vehicle control-treated cells (DMSO).

#### 4.2.5. Plasmids and Generation of the Stable Cell Lines

The DNA of the human rhodopsin gene (hRHO, NCBI Ref. Sequence: NM_000539.2), purchased from Genscript (Piscataway, NJ, USA), was cloned into pcDNA™5/FRT/TO (Flp-In™ T-REx™ Core kit, Thermo Fisher Scientific (Waltham, MA, USA) through Gibson assembly according to the manufacturer’s instructions (Gibson Assembly^®^ Master Mix, New England Biolabs, Ipswich, MA, USA) with the addition of 6× histidine at the C-terminus (hRHO WT His-Tag). 5′-TTGGTACCGAGCTCGGATCCGCCACCATGAAT-3′ and 5′-TCAATGGTGATGGTGATGATGGGCCGGGGCCACCTGG-3′ were used to PCR amplify the hRHO gene, 5′-TCTGTGCCATTCATGGTGGCGGATCCGAGCTCGGTACCA-3′ and 5′- CATCATCACCATCACCATTGACAGATATCCAGCACAGTGGCGGCC-3′ the vector (underlined codons were used to introduce the His-Tag). Site-directed mutagenesis to introduce P23H mutation (hRHO P23H His-tag) was performed with the QuikChange Lightning kit (Agilent) according to the manufacturer’s instructions, using the following primers: 5′-TGTGGTACGCAGCCACTTCGAGTACCCAC-3′ and 5′-GTGGGTACTCGAAGTGGCTGCGTACCACA-3′ (mutated codon is underlined). All primers were purchased from IDT (Integrated DNA Technologies, IDT, Coralville, IA, USA). All plasmid constructs were verified through sequencing (Eurofins Genomic UK, Wolverhampton, UK).

The tetracycline-inducible expression of hRHO P23H mutant was established in U2OS using plasmids from the Flp-In™ T-REx™ Core kit (Thermo Fisher Scientific, Waltham, MA, USA). Briefly, U2OS cells transfected with 1 μg pcDNA6/TR plasmid and Fugene HD (1:3 DNA:FUGENE HD ratio, Promega) were subsequently cultured in media containing 10 µg/mL blasticidin (Thermo Fisher Scientific), selecting the clone that exhibited the lowest levels of basal transcription of the tetracycline repressor (TetR) but the highest levels of transcription after addition of tetracycline to the media. Subsequently, the clone was transfected with 1 μg of pcDNA5/FRT/TO hRHO P23H and Fugene HD and selected with 50 ug/mL Zeocin (Thermo Fisher Scientific). Inducible expression of hRHO P23H was checked through immunofluorescence microscopy.

#### 4.2.6. Immunofluorescence Microscopy

Cells were plated on 13 mm polylysine-treated round glass coverslips and let to adhere for at least 4 h. Media was replaced with media containing 1 µg/mL of tetracycline and 5 µM of 9-*cis*-retinal or compound (**2**, **8**, **11**, **13**, **18** and **20** at 10 µM final concentration, **CF35EsC**, **CF35Es** and the remaining compounds at 20 µM final concentration, DMSO not exceeding 0.1%). **CF35EsC** and **CF35Es** were used as a positive control. Cells were incubated overnight in the dark to allow the tetracycline-induced expression of rhodopsin. On the following day, under dim red light conditions, cells were washed twice in PBS and fixed for 25 min using a solution of methanol-free 4% paraformaldehyde, then washed in PBS before blocking 1 h with TBS blocking buffer (LI-COR, Lincoln, NE, USA) solution followed by incubation with RET-P1 antibody (Invitrogen, 1:500, Carlsbad, CA, USA) in TBS buffer and, subsequently, with anti-mouse IgG (H + L), F(ab’) 2 Fragment Alexa Fluor ^®^ 555 Conjugate (Cell Signalling Technologies, 1:1000, TBS buffer, Danvers, MA, USA) for 1 h. Coverslips were then carefully washed before permeabilising cell membranes with a solution of Triton 0.1% for 20 min. Anti-His Tag (Amgen, 1:500, TBS buffer, Thousand Oaks, CA, USA) was then incubated with the cells overnight followed by a 1 h incubation with an anti-mouse IgG (H + L), F(ab’)2 Fragment Alexa Fluor^®^ 488 Conjugate antibody (Cell Signalling Technologies, 1:1000, TBS buffer). Nuclei were stained with DAPI before mounting the coverslips on glass microscope slides. Immunofluorescence images were captured with an Olympus BX50 epifluorescence microscope, Southend-on-Sea, UK, (from 3 independent experiments). Gain and exposure times for 488 nm and 555 nm channels were maintained constant across all coverslips from the same experiment, which always included DMSO and 9-*cis*-retinal as controls. Images were processed with ImageJ software: the contrast was enhanced in the 405 nm channel for clarity, whilst no manipulations were made to the channels relevant in the experiments (488 and 555 nm). RGB colours were assigned to each channel (blue to 405 nm, green to 488 nm and red to 555 nm) and images were merged with the software in-built function.

## Figures and Tables

**Table 1:** Calculated ligand interaction energies for the compounds analysed after the molecular dynamic simulations.

Compound	∆G_binding_ (kJ/mol) ^a^ ± SD
**6**	**−67.08 ± 9.93**
**8**	**−73.18 ± 6.43**
**9**	n.c.
**13**	−68.12 ± 5.76
**17**	−63.93 ± 5.79
**20**	**−77.93 ± 5.71**
**21**	n.c.
**22**	n.c.
**23**	**−74.30 ± 5.93**
**CF35EsC**	−60.53 ± 5.62
**CF35Es**	−64.25 ± 4.51

^a^ ΔG_binding_ average values calculated as the mean from three independent molecular dynamic (MD) simulations (triplicate) for each compound. For each replicate, the ΔG_binding_ value was calculated excluding the first 40 ns of MD, in which the system protein–ligand reached stability, except for compounds **9**, **21** and **22**. The ΔG_binding_ values were extracted every 0.33 ns for each replicate. Standard deviation (SD) is reported. n.c. = not calculated: simulation systems that are not able to equilibrate due to the presence of the compounds are considered not reliable for a rigorous analysis and ΔG_binding_ calculation. New compounds defined as hits by the isorhodopsin regeneration assay are printed in bold.

## Supplementary Materials

The following are available online. Figure S1: Synthetic preparation of the two reference compounds **CF35EsC** and **CF35Es**; Table S1: Effect of compound **1**–**24** on rate constant (K) of bovine isorhodopsin regeneration; Figure S2: Plots of C-alpha RMSD (Å) values against simulation time for rhodopsin; Figure S3: Plots of C-alpha RMSD (Å) values against simulation time for protein–ligand complexes; Figure S4: Plots of C-alpha RMSD (Å) values against simulation time for not equilibrated protein–ligand systems; Figure S5: Subcellular localisation of C-terminus His-tagged human rod opsin wild type (hRHO WT His-Tag) in transiently transfected U2OS cells.
