# Supplementary material for: Computational Studies towards the Identification of Novel Rhodopsin-Binding Compounds as Chemical Chaperones for Misfolded Opsins
Source: Molecules. 2020 Oct 23;25(21):4904. doi: 10.3390/molecules25214904 (PMC7660337; doi:10.3390/molecules25214904)
Supplement: Supplementary file 1 [file molecules-25-04904-s001.pdf]

Computational studies towards the identification of novel rhodopsin-binding compounds as chemical chaperones for misfolded opsins

Gaia Pasqualetto[1], Martin Schepelmann[2,3], Carmine Varricchio[1], Elisa Pileggi[1], Caroline Khogali[3], Sian Morgan[3,4], Ian Boostrom[3], Malgorzata Rozanowska[3,4], Andrea Brancale[1], Salvatore Ferla[5], Marcella Bassetto[6].

[1]School of Pharmacy and Pharmaceutical Sciences, Cardiff University, Cardiff, United Kingdom.

[2]Institute of Pathophysiology and Allergy Research, Medical University of Vienna, Vienna, Austria.

[3]School of Optometry and Vision Sciences, Cardiff University, Cardiff, United Kingdom.

[4]Cardiff Institute for Tissue Engineering and Repair (CITER), Cardiff University, Cardiff, United Kingdom.

[5]Swansea University Medical School, Swansea, United Kingdom.

[6]Department of Chemistry, Swansea University, Swansea United Kingdom.

Correspondence and requests for material s should be addressed to Professor A. Brancale (email: brancalea@cardiff.ac.uk).

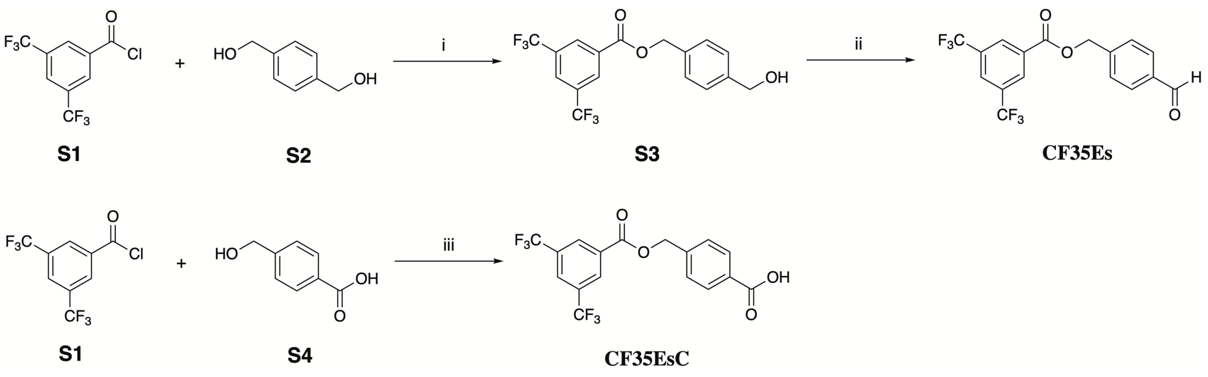

Figure S1: Synthetic preparation of the two reference compounds CF35Es and CF35EsC.
Reagents and conditions: i) cat. DMAP, Et_3N, anhydrous DCM, 0 °C to 50 °C, o.n. (52%); ii) Dess-Martin periodinane, anhydrous DCM, r.t., 4 h (99%); iii) cat. DMAP, Pyridine, 0 °C to r.t., 16 h (78%).

4-Formylbenzyl 3,5-bis(trifluoromethyl)benzoate (**CF35Es**)
Commercial acyl chloride **S1** (0.33 mL, 1.81 mmol) was added dropwise to a stirring solution of para-di-benzyl alcohol **S2** (0.5 gr, 3.62 mmol), triethylamine (0.8 mL, 5.43 mmol) and dimethylaminopyridine (DMAP, 0.54 mmol) in 7 mL anhydrous dichloromethane (DCM) at 0 °C under a nitrogen atmosphere. The reaction was allowed to warm to r.t., then heated at 50 °C and stirred overnight. The mixture was then cooled to r.t., diluted with DCM (25 mL), washed with a 2M aqueous HCl solution (2x 30 mL), dried over $MgSO_4$ and concentrated under *vacuum*. The crude residue was purified by automated flash column chromatography on silica gel (n-hexane:EtOAc 100:0 to n-hexane:EtOAc 0:100 in 10 CV) to afford intermediate **S3** as a white solid in 52% yield. **S3** (0.29 gr, 0.76 mmol) was then dissolved in 7 mL anhydrous DCM under a nitrogen atmosphere. Dess-Martin periodinane (DMP, 0.39 gr, 0.91 mmol) was then added in one portion, and the reaction was stirred at r.t. for 4 hours. The mixture was then diluted with EtOAc (25 mL) and washed with saturated aqueous NaHCO3 solution (25 mL) and brine (20 mL). The organic layer was dried over $MgSO_4$ and concentrated under *vacuum*. The crude residue was then purified by automated flash column chromatography on silica gel (n-hexane:EtOAc 100:0 to n-hexane:EtOAc 40:60 in 10 CV) to afford the pure title compound as a white solid. Analytical data obtained are in accordance with literature values [S1].
4-((3,5-bis(Trifluoromethyl)benzoyloxy)methyl)benzoic acid (**CF35EsC**)
Commercial acyl chloride **S1** (0.33 mL, 1.81 mmol) was added dropwise to a stirring solution of *para*-substituted benzyl alcohol **S4** (0.28 gr, 1.81 mmol) in pyridine (5 mL) at 0 °C under a nitrogen atmosphere. DMAP (0.54 mmol) was then added and the reaction was allowed to warm to r.t., then stirred for 16 hours. The mixture was then diluted with EtOAc (25 mL) and extracted with 2M aqueous HCl solution (2x 25 mL). The organic layer was dried over $MgSO_4$ and concentrated under *vacuum*. The crude residue was then purified by automated flash column chromatography on silica gel (n-hexane:EtOAc 100:0 to n-hexane:EtOAc 0:100 in 10 CV) to afford the pure title compound as a white solid. Analytical data obtained are in accordance with literature values [S1].

Table S1: Effect of compound **1-24** (compound concentration 10 times 9-*cis*-retinal) on rate constant (**K**) of bovine isorhodopsin regeneration. After bleaching, compounds were pre-incubated for 30 min followed by addition of 9-*cis*-retinal. **β-ionone**, **CF35EsC** and **CF35Es** were used as positive controls. Compounds were considered 'hit' when K ≥ 10% decreased or ≥ 20% increased compared to DMSO. Bars represent mean ± SEM of pooled data (at least three independent measurements), No data were obtained for **5**, **11** and **12** due to solubility problems at the tested concentration.

**Compound**	**Rate constant (K) (min^{-1}) ±SEM**	**Compound**	**Rate constant (K) (min^{-1}) ±SEM**
**1**	0.64±0.09	**17**	0.46±0.03
**2**	0.54±0.09	**18**	0.52±0.05
**3**	0.55±0.08	**19**	0.43±0.07
**4**	0.56±0.06	**20**	0.35±0.02
**6**	0.44±0.01	**21**	0.48±0.04
**7**	0.61±0.08	**22**	0.66±0.09
**8**	0.40±0.02	**23**	0.37±0.04
**9**	0.49±0.01	**24**	0.43±0.05
**10**	0.56±0.06	**DMSO**	**0.48±0.01**
**13**	0.46±0.04	**β-ionone**	0.20±0.01
**14**	0.44±0.04	**CF35EsC**	0.61±0.09
**15**	0.46±0.04	**CF35Es**	0.42±0.05
**16**	0.47±0.05		

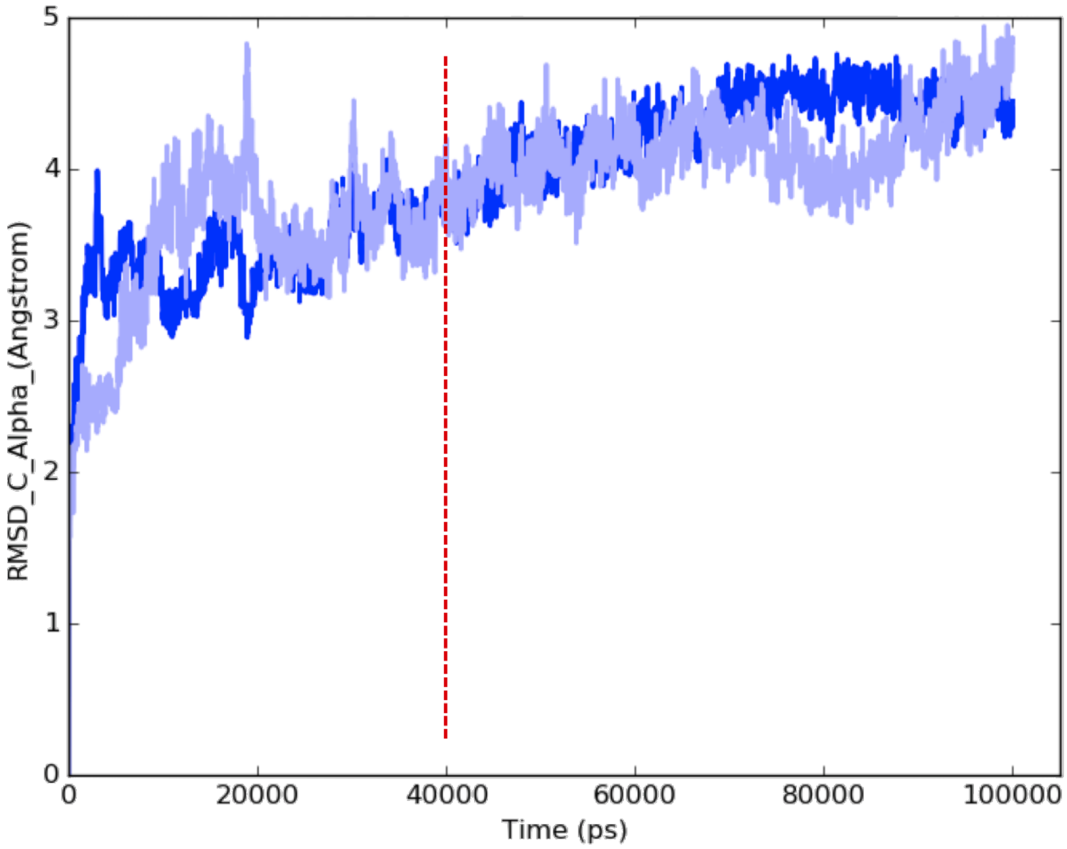

Figure S2: Plots of C-alpha RMSD (Å) values against simulation time for rhodopsin. After 40 ns of equilibration (red dotted line), the rhodopsin-11-*cis*-retinal complex reaches stability and it is characterised by a small RMSD variation for the rest of the simulation. On the contrary, the free rhodopsin is not able to reach stability after 40 ns and the RMSD value is still growing toward the end of the simulation Only one replicate is shown as example. Colour legend: rhodopsin-11-*cis*-retinal complex (blue), free rhodopsin (lilac).

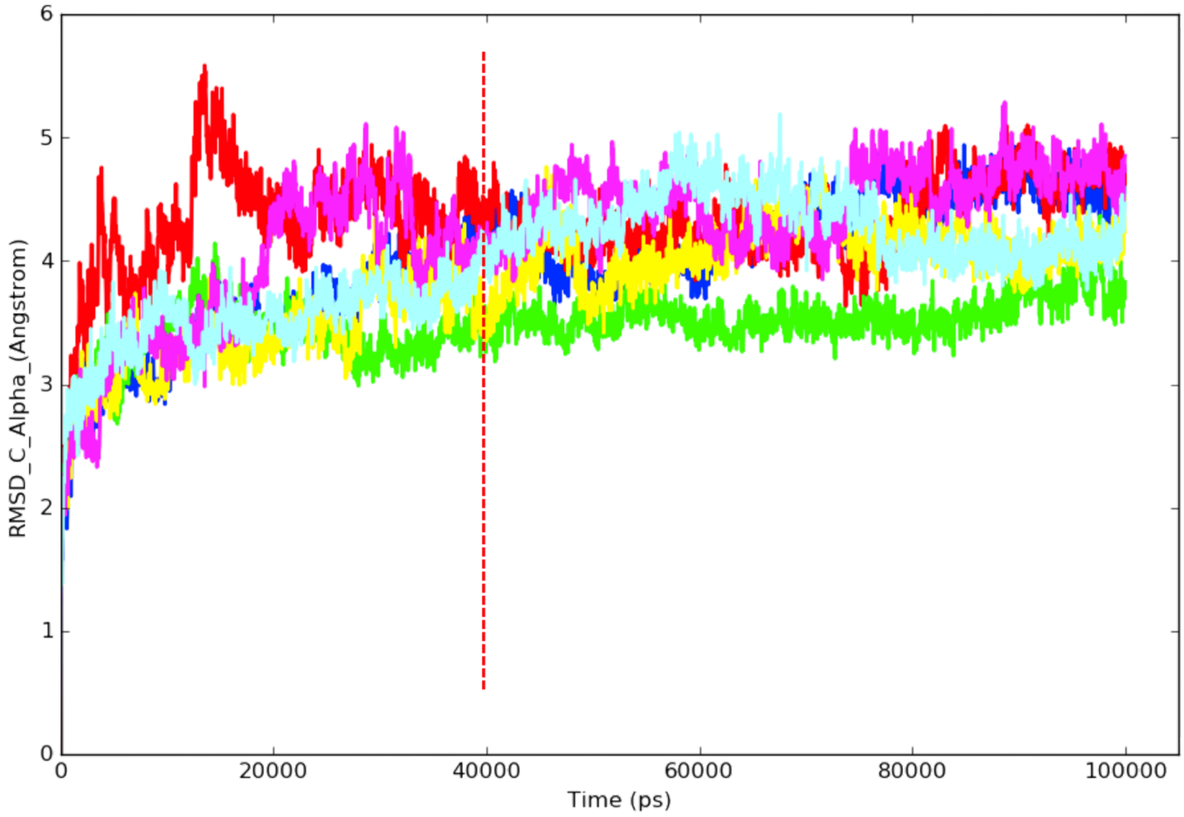

Figure S3: Plots of C-alpha RMSD (Å) values against simulation time for protein-ligand complexes. After 40 ns of equilibration (red dotted line), in each complex, the protein reaches stability and it is characterised by a small RMSD variation for the rest of the simulation. Only one replicate is shown for each protein-ligand complex as example. Colour legend: **6**-rhodopsin (blue), **8**-rhodopsin (red), **13**-rhodopsin (green), **17**-rhodopsin (yellow), **20**-rhodopsin (violet), **23**-rhodopsin (light blue).

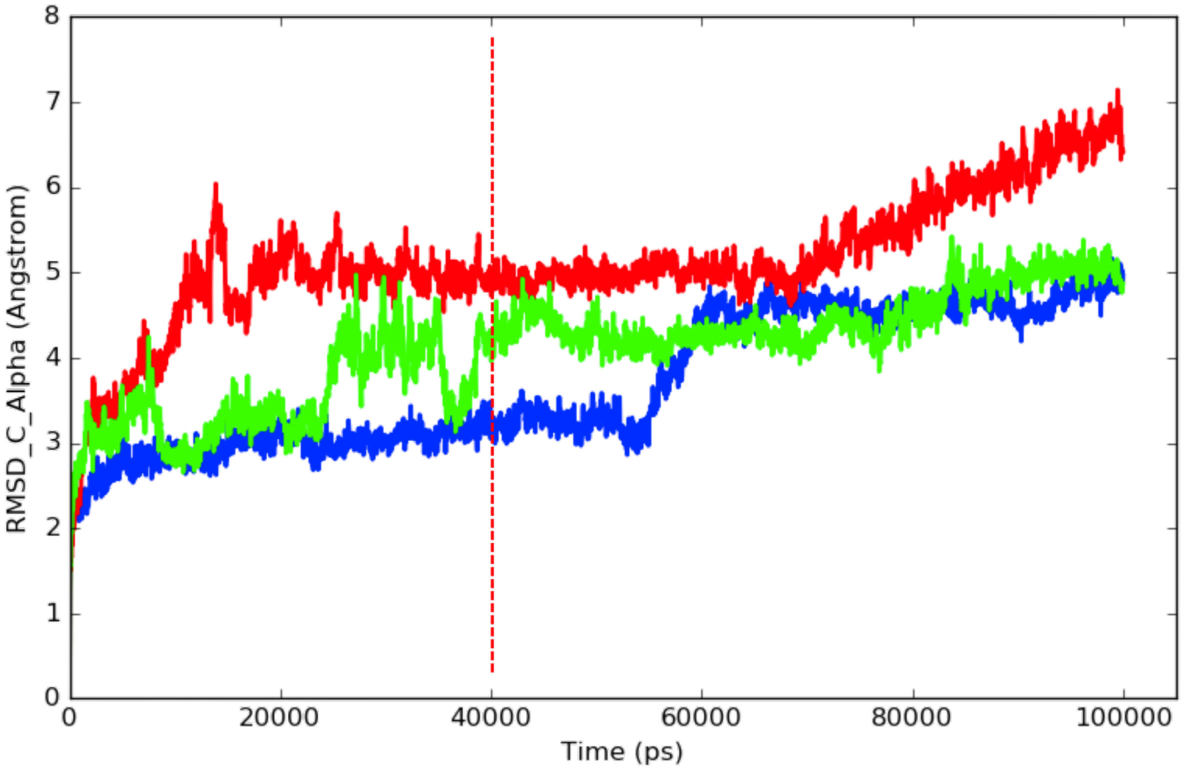

Figure S4: Plots of C-alpha RMSD (Å) values against simulation time for not equilibrated protein-ligand systems. After 40 ns (red dotted line, the three different complexes were not able to equilibrate and the protein RMSD is still growing toward the end of the simulation. Only one replicate is shown for each protein-ligand complex as example. Colour legend: **9**-rhodopsin (blue), **21**-rhodopsin (red), **22**-rhodopsin (green).

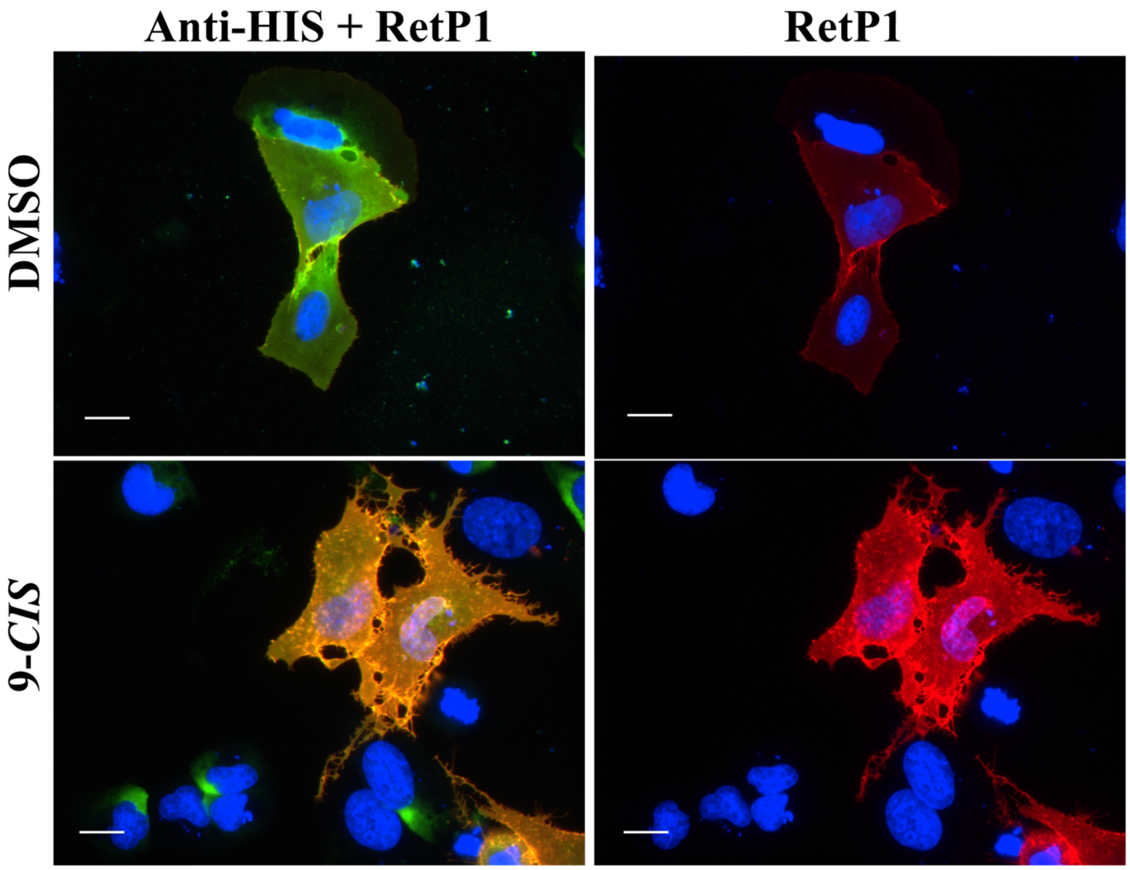

Figure S5: Subcellular localization of C-terminus His-tagged human rod opsin wild type (hRHO WT His-Tag) in transiently transfected U2OS cells. Representative images were taken in absence or presence of 5 µM 9-*cis*-retinal (9-CIS). Anti-His/RetP1: merged image of RetP1 staining (red) and Anti-His Tag (post-permeabilisation; green). RetP1: anti-rhodopsin antibody (N-terminus, red) staining only protein exposed on cell membrane. Nuclei stained with DAPI (blue). Scale bars: 10 µm.

References

S1. Ohgane, K. et al. Retinobenzaldehydes as proper-trafficking inducers of folding-defective P23H rhodopsin mutant responsible for retinitis pigmentosa. *Bioorg. Med. Chem.* **2010**; 18, 7022-7028.
